# Progress and Prospects of Triazoles in Advanced Therapies for Parasitic Diseases

**DOI:** 10.3390/tropicalmed10050142

**Published:** 2025-05-20

**Authors:** Jaime A. Isern, Renzo Carlucci, Guillermo R. Labadie, Exequiel O. J. Porta

**Affiliations:** 1 Leibniz-Forschungsinstitut für Molekulare Pharmakologie (FMP), Robert-Rössle-Str. 10, 13125 Berlin, Germany; isern@fmp-berlin.de; 2 Instituto de Química Rosario, Universidad Nacional de Rosario, CONICET, S2002LRK Rosario, Argentina; carlucci@iquir-conicet.gov.ar (R.C.); labadie@iquir-conicet.gov.ar (G.R.L.); 3 Departamento de Química Orgánica, Facultad de Ciencias Bioquímicas y Farmacéuticas, Universidad Nacional de Rosario, S2002LRK Rosario, Argentina; 4 UCL School of Pharmacy, Faculty of Life Sciences, University College London (UCL), 29-39 Brunswick Square, London WC1N 1AX, UK

**Keywords:** triazoles, parasitic diseases, click chemistry, antiparasitic, malaria, anthelmintic, neglected tropical diseases, Chagas disease, leishmaniasis, combination therapy

## Abstract

Parasitic diseases represent a severe global burden, with current treatments often limited by toxicity, drug resistance, and suboptimal efficacy in chronic infections. This review examines the emerging role of triazole-based compounds, originally developed as antifungals, in advanced antiparasitic therapy. Their unique structural properties, particularly those of 1,2,3- and 1,2,4-triazole isomers, facilitate diverse binding interactions and favorable pharmacokinetics. By leveraging innovative synthetic approaches, such as click chemistry (copper-catalyzed azide–alkyne cycloaddition) and structure-based design, researchers have repurposed and optimized triazole scaffolds to target essential parasite pathways, including sterol biosynthesis via CYP51 and other novel enzymatic routes. Preclinical studies in models of Chagas disease, leishmaniasis, malaria, and helminth infections demonstrate that derivatives like posaconazole, ravuconazole, and DSM265 exhibit potent in vitro and in vivo activity, although their primarily static effects have limited their success as monotherapies in chronic cases. Combination strategies and hybrid molecules have demonstrated the potential to enhance efficacy and mitigate drug resistance. Despite challenges in achieving complete parasite clearance and managing potential toxicity, interdisciplinary efforts across medicinal chemistry, parasitology, and clinical research highlight the significant potential of triazoles as components of next-generation, patient-friendly antiparasitic regimens. These findings support the further optimization and clinical evaluation of triazole-based agents to improve treatments for neglected parasitic diseases.

## 1. Introduction

**Figure 1:**
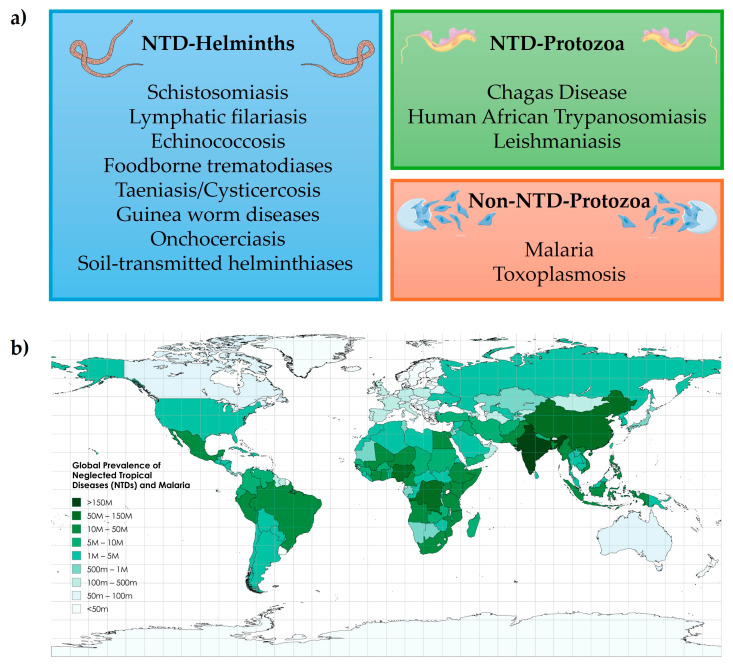
(**a**) Classification of certain parasitic diseases by etiological agent: helminthic vs. protozoan infections (and their NTD status). (**b**) Global prevalence of NTDs and malaria, collectively affecting over 1.1 billion people worldwide in 2021 [10].

Parasitic diseases caused by protozoa and helminths impose a significant health and economic burden worldwide (Figure 1). Neglected tropical diseases (NTDs) alone required interventions for approximately 1.62 billion people in 2022 [1], with an estimated cost of USD 0.5 billion per year in lower-middle-income countries and accounting for roughly 519 million disability-adjusted life years (DALYs) between 2015 and 2030 if left unchecked [2]. Malaria (caused by *Plasmodium* protozoa) kills roughly 600,000 people annually despite global control efforts [3], and its global expenditure exceeded USD 4.3 billion recently; however, in sub-Saharan Africa, it still slows GDP-per-capita growth by about 1.3 % annually and imposes nearly USD 12 billion in economic losses each year through healthcare costs and lost productivity [4]. Schistosomiasis reduces agricultural output by an average of 6.6% in affected areas and up to 32% for households in high-intensity clusters, undermining food security and livelihoods [5]. Likewise, leishmaniasis outbreaks have disrupted economic development programs in regions such as the Amazon basin, Morocco, and Saudi Arabia by diverting scarce resources to control efforts and deepening poverty cycles in vulnerable communities [6]. Although classically confined to tropical areas, NTDs are increasingly encountered in non-endemic regions. For instance, an estimated 68,000–122,000 people living in Europe and approximately 300,000 people living in the United States harbor chronic *T. cruzi* infections (acquired via migration, congenital transmission, or transfusion) yet remain largely underdiagnosed [7,8]. Similarly, cutaneous and visceral leishmaniasis cases (both imported and autochthonous) have been documented in southern Europe and the southern United States, driven by travel, migration, and changing sand fly distributions [9].

The efficacy of many existing antiparasitic drugs has declined due to issues such as drug resistance, suboptimal safety, and incomplete cures in chronic infections [11]. For example, frontline treatments for Chagas disease (*Trypanosoma cruzi* infection) and leishmaniasis (*Leishmania* spp. infection) rely on old drugs, such as nifurtimox, benznidazole, or antimonial compounds, which exhibit high toxicity and variable effectiveness [12]. Meanwhile, *Plasmodium falciparum* (the primary agent of severe malaria) has developed resistance to multiple drug classes, such as chloroquine, as well as partial resistance to artemisinin [13]. These challenges drive the search for new or repurposed therapies with novel mechanisms of action [14].

**Figure 2:**
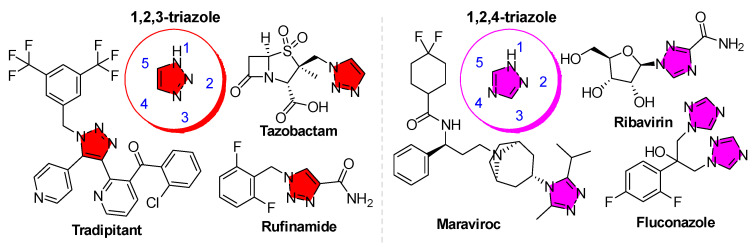
Representative drug molecules incorporating 1,2,3-triazole (in red) and 1,2,4-triazole (in magenta) heterocycles.

Heterocyclic compounds featuring azole rings have long been important in anti-infective therapy. Triazoles are five-membered aromatic heterocycles containing three nitrogen atoms. They exist as two constitutional isomers, 1,2,3-triazoles and 1,2,4-triazoles, each of which can adopt three tautomeric forms (*1H*, *2H*, and *4H* for 1,2,3-triazoles; *1H*, *3H*, and *4H* for 1,2,4-triazoles), yielding six distinct isomeric structures. This review focuses on the classification of the two constitutional isomers (i.e., 1,2,3- and 1,2,4-triazoles), both of which serve as valuable pharmacophores (Figure 2). Critically, the triazole scaffold is associated with broad-spectrum bioactivity and favorable drug-like properties, such as good oral bioavailability, high metabolic stability, and low toxicity [15]. Its unique structure enables the formation of multiple non-covalent interactions (hydrogen bonds, π-π stacking, dipole interactions, etc.) with biological targets [16]. In human medicine, 1,2,4-triazole rings are found in a variety of potent drugs, most prominently the triazole antifungals (e.g., fluconazole, itraconazole, posaconazole), which revolutionized the treatment of systemic mycoses [17]. In particular, the 1,2,3-triazole is a moderately dipolar, aromatic ring capable of hydrogen bonding and π interactions, which allows it to stabilize protein–ligand complexes through diverse binding modes [18]. Likewise, 1,2,4-triazoles have proven to be privileged structures in medicinal chemistry. They appear as the core of many drugs across different therapeutic areas (antifungal azoles, antiviral ribavirin, anticancer letrozole, etc.), attesting to their versatility [19].

Triazole compounds can target parasite pathways analogous to those they disrupt in fungi, which is of particular relevance to antiparasitic applications. A prime example is parasitic protozoa’s dependence on sterol biosynthesis. Many protozoan parasites (e.g., *T. cruzi* and *Leishmania* spp.) synthesize sterols (ergosterol or analogs) for their cell membranes, using a cytochrome P450 14α-demethylase (CYP51) enzyme just as fungi do [20]. Clinical antifungal triazoles, mainly 1,2,4-triazoles, inhibit fungal CYP51 by blocking the conversion of lanosterol to ergosterol, thereby disrupting cell membrane integrity [21]. The triazole ring’s lone pair of electrons on the N^4^ (for 1,2,4-triazoles) coordinates strongly to the heme iron of CYP51, inhibiting the enzyme [22]. This mechanism can likewise kill or stunt parasites that rely on ergosterol, making antifungal triazoles attractive candidates for repurposing to treat diseases such as Chagas [23] and leishmaniasis [24]. Indeed, broad-spectrum azoles like posaconazole have shown strong activity against *T. cruzi* in vitro and in mouse models, motivating clinical trials involving the treatment of Chagas disease [25].

Another reason triazoles are of great interest in advanced antiparasitic therapy is the development of powerful synthetic methods to create and modify these molecules. The rise of “click chemistry”, in particular the Cu(I)-catalyzed azide–alkyne cycloaddition (CuAAC), described by Sharpless and colleagues, allows medicinal chemists to synthesize 1,2,3-triazoles in high yield under mild conditions [26]. This reaction, a prototypical click reaction, can rapidly generate exclusively 1,4-disubstituted 1,2,3-triazoles from an azide and an alkyne and has had a profound impact on drug discovery [27]. For instance, it enables the modular assembly of triazole-based hybrid molecules, in which a 1,2,3-triazole linkage connects two bioactive pharmacophores [28]. Such hybrids have been pursued to obtain multi-functional antiparasitic agents that can hit multiple targets or overcome drug resistance. Moreover, the 1,2,3-triazole moiety not only can act as a linker but also as a binder, thereby enhancing the interactions between protein and ligands [29]. The 1,2,3-triazole ring itself is chemically stable (resistant to hydrolysis, oxidation, and metabolic degradation), making it an ideal linker or scaffold in drug design. Given that the triazole motif exhibits chemical robustness, ease of synthesis, and polypharmacology, it poses as a promising cornerstone for next-generation antiparasitic drugs [30,31].

It is important to distinguish that the term “azoles” in antiparasitic therapy encompasses not only triazoles but also benzimidazoles, a distinct chemotype featuring a fused benzene–imidazole ring, which are widely used as anthelmintics [32]. Benzimidazole drugs, such as albendazole and mebendazole, are essential for treating worm infections (e.g., soil-transmitted helminths, filariasis), but they differ from triazoles in both structure and mechanism of action, as they target parasite tubulin rather than sterol biosynthesis [33]. This review focuses exclusively on triazoles and their role in advanced therapies for protozoan parasitic diseases and helminth infections. We discuss their structural and pharmacological properties that are relevant to antiparasitic activity, examine their application in treating diseases such as American trypanosomiasis (Chagas disease), leishmaniasis, human African trypanosomiasis (HAT), malaria, and schistosomiasis, among others, considering both monotherapy and combination approaches. Challenges related to efficacy, drug resistance, and safety are evaluated, highlighting current medicinal chemistry strategies to address these issues. We also assess future directions, from ongoing clinical trials to innovative hybrid molecules designed using click chemistry. This review critically evaluates the literature primarily from the past decade (2015–2025), a period characterized by notable advances in synthetic methodologies and drug repurposing strategies involving triazole derivatives. While previous reviews have extensively covered the use of azoles in antifungal therapies [17,34,35], comprehensive analyses specifically focused on their emerging role in antiparasitic therapies are limited. Thus, our goal is to fill this gap by thoroughly discussing recent progress and persisting challenges in developing triazole-based antiparasitic agents, with emphasis on structural optimization, pharmacological profiles, and novel strategies to overcome current therapeutic limitations and drug resistance.

## 2. Structural and Mechanistic Features of Triazoles in Antiparasitic Drug Design

### 2.1. Triazole Isomers and Pharmacophore Properties

**Figure 3:**
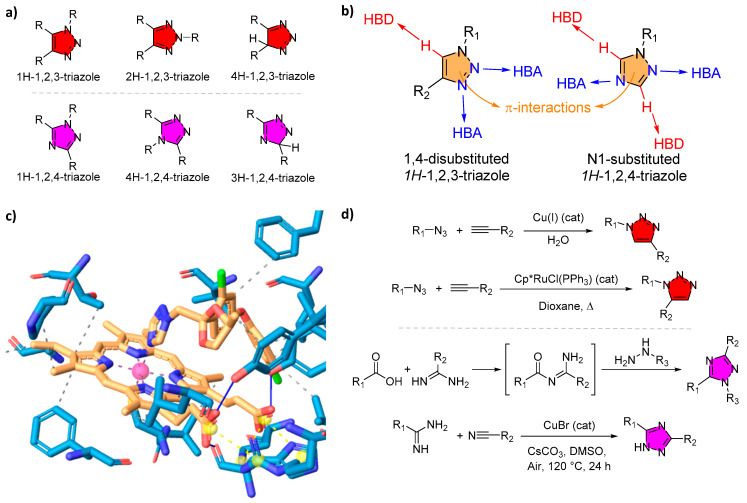
Triazole isomers, properties, and synthesis. (**a**) Chemical structures of six triazole isomers. (**b**) Illustration of hydrogen bond donor (HBD) and hydrogen bond acceptor (HBA) properties of triazoles, along with potential π interactions. (**c**) Representative metal-binding mode of a triazole ligand within a CYP51, depicted with PLIP [57] (PDB ID: 5EAH). (**d**) Common synthetic routes for triazole formation.

As mentioned above, triazoles exist in two main isomeric forms (1,2,4-triazoles and 1,2,3-triazoles), which differ in the arrangement of nitrogen atoms in the ring (Figure 3a). Both are planar aromatic heterocycles with six π-electrons conferring significant stability [36]. The aromaticity and high nitrogen content make triazoles electron-rich, polar, and able to engage in hydrogen bonding as both acceptors and (for some ring tautomer and substitution patterns) donors. The 1,2,4-triazole is the scaffold found in almost all current azole medications, including antifungal drugs and others, mainly with a N^1^-substituted ring pattern. On the other hand, 1,2,3-triazoles are not yet common in approved drugs but are extensively used in chemical biology and drug discovery, mainly with a 1,4-disubstituted ring pattern (*vide infra*). In fact, there is only one approved 1,4-disubstituted-1,2,3-triazole, named Rufinamide, which is used for anti-seizure treatment [37]. They act as rigid, metabolically stable linkers or core structures that can serve as bioisosteres of amide bonds or phenyl rings in drug molecules without disrupting binding. The 1,2,3-triazole ring exhibits a rich interaction profile with proteins; crystallographic data mining has shown that 1,2,3-triazole rings participate in classical hydrogen bonds, as well as π-π stacking and cation–π interactions with amino acid residues (Figure 3b) [38]. These interactions allow 1,2,3-triazoles to function as more than just linkers (they can directly contribute to affinity and selectivity in ligand binding). This remains an active area of research as scientists continue to optimize triazole-containing drug candidates by studying the ring’s orientation and interactions within target enzyme pockets.

### 2.2. Coordination with Metals and the Inhibition of CYP Enzymes

A defining mechanistic feature of triazoles (especially 1,2,4-triazoles) is their ability to coordinate to metalloproteins, such as cytochrome P450 enzymes (CYPs). Triazole antifungals exploit this by binding tightly to the heme iron of CYP51 (lanosterol 14α-demethylase) in fungi (Figure 3c) [39]. The triazole’s basic nitrogen displaces the iron-bound water, locking the enzyme in an inhibited state and blocking sterol demethylation [40]. In parasitic protozoa, sterol 14α-demethylase is likewise essential and is sufficiently similar to the fungal enzyme that many antifungal azoles inhibit it. For instance, ketoconazole, fluconazole, itraconazole, posaconazole, and ravuconazole all inhibit *T. cruzi* CYP51 and have antiproliferative effects on the parasite [41]. High-resolution structures reveal that posaconazole can bind the *T. cruzi* enzyme in a mode virtually identical to how ketoconazole binds human CYP51, reflecting the conserved active site geometry [25]. This conservation explains why broad-spectrum azoles are active against trypanosomatid parasites but also suggests that achieving selectivity (parasite vs. host) might be challenging, a topic we revisit under resistance and toxicity considerations (*vide infra*). Beyond CYP51, triazole-containing compounds can target other parasite metalloproteins or enzymes. For example, 1,2,4-triazoles have been incorporated into iron-chelating drugs and NO-donor hybrids to leverage metal-binding in antiparasitic mechanisms [42]. The key point is that the coordination chemistry of the triazole ring provides a powerful means to inhibit metalloenzymes that are essential for parasite survival.

### 2.3. “Click Chemistry” and Derivatization

While numerous reviews have focused on the synthesis of 1,2,3-triazoles and 1,2,4-triazoles (Figure 3d) [16,36,43,44,45], this review specifically highlights the landmark discovery of click chemistry and its transformative impact on the development of novel bioactive structures. The ease of synthesizing and modifying triazoles via click chemistry has dramatically expanded the chemical space of triazole derivatives in terms of testing against parasites. The CuAAC, first popularized by Sharpless in 2001, is high-yielding and compatible with a wide range of functional groups [26]. It has become a staple method to create libraries of 1,4-disubstituted 1,2,3-triazole compounds. Notably, CuAAC is modular: one can vary the azide part (which contributes three ring nitrogens and an N^1^ adjacent substituent) and the alkyne part (contributing the two carbon of the ring and the other C^4^ substituent) independently to quickly assemble diverse analogs. This has enabled medicinal chemists, for example, to generate triazole hybrids that combine known pharmacophores with a triazole linker [46]. In antiparasitic research, such hybrids include quinoline–triazole conjugates (linking a 4-aminoquinoline, like chloroquine, with another moiety), triazole–artemisinin hybrids, triazole–benzimidazole hybrids, and many others [47]. The goal of these hybrids is often to achieve dual action (e.g., one part of the molecule might target heme detoxification in malaria parasites while the triazole part targets a different enzyme) or to improve pharmacokinetic properties. Some 1,2,3-triazole hybrids, such as triazole-linked quinoline hybrids (*vide infra*), have demonstrated the ability to hit multiple stages of a parasite’s lifecycle, an attractive feature for diseases like malaria that have distinct blood and liver stages [48]. Recently, a straightforward copper-catalyzed one-pot synthesis of 3,5-disubstituted-1,2,4-triazoles from amides and nitriles via a cascade addition–oxidation–cyclization reaction has been developed [49].

Furthermore, click chemistry can be performed in a biocompatible, metal-free manner (e.g., using strain-promoted cycloaddition with cyclooctynes) [50], which has facilitated attaching triazoles to biomolecules and surfaces for targeted drug delivery. Additionally, researchers have used 1,2,3-triazole linkages to attach drugs onto nanoparticles or hydrogels for controlled release to infection sites, taking advantage of triazole stability in physiological conditions [51]. While these applications are still experimental, they represent “advanced therapies” in the sense that they feature novel delivery systems that could improve antiparasitic treatment efficacy and safety.

### 2.4. Pharmacokinetics and Metabolic Stability

Triazoles generally impart favorable pharmacokinetic properties to drug molecules. Many triazole drugs have long half-lives in humans (e.g., fluconazole: ~30 h, posaconazole: ~35 h), allowing for once daily or less frequent dosing [52]. The metabolic inertness of the triazole ring (particularly, 1,2,3-triazoles that resist enzymatic cleavage) helps ensure that the drug’s core remains intact, although metabolic modifications can still occur on its substituents. One caveat is that some triazoles can inhibit human drug-metabolizing CYP enzymes (like CYP3A4), leading to drug–drug interactions [53]. This effect is well known in azole antifungals. For example, ketoconazole, an imidazole derivative, strongly inhibits CYP3A4 and was found to cause dangerous increases in other drug levels (e.g., cyclophosphamide, cyclosporine, imatinib, bosutinib, among others), contributing to reducing its use as a systemic antifungal [54]. This is particularly relevant in co-endemic regions where polypharmacy is common, as CYP3A4 inhibition can alter the pharmacokinetics of co-administered therapies for HIV, tuberculosis, and other parasitic diseases, thereby increasing the risk of adverse drug–drug interactions [55]. Newer triazoles exhibit improved specificity; isavuconazole, an advanced 1,2,4-triazole, was designed to exhibit a less complex interaction profile and more predictable pharmacokinetics. Indeed, isavuconazole has fewer problematic interactions and lacks QT prolongation (the interval between the onset of the Q wave and the end of the T wave on an electrocardiogram), which is a side effect seen with other 1,2,4-triazoles, such as voriconazole [56], reflecting how triazole drug design has evolved to optimize safety while retaining potency. As a result, the triazole scaffold emerges as a particularly valuable motif, offering strong target binding (through multi-point interactions and metal coordination), along with synthetic flexibility and pharmacokinetic robustness. These features underpin the growing role of triazoles in addressing unmet needs in parasitic disease therapy.

## 3. Current Progress of Triazole-Based Therapies for Protozoan Parasitic Diseases

Triazole-based compounds have emerged as promising candidates for treating protozoan parasitic diseases due to their robust in vitro and in vivo activities and favorable drug metabolism and pharmacokinetics (DMPK) profiles. Recent studies have focused on refining their structural features to improve potency and selectivity against various protozoan pathogens, thereby addressing the challenge of drug resistance [47,48]. In the following sections, we detail the latest advances in triazole-based therapies and explore their potential in transforming the treatment landscape for these infections.

### 3.1. American Trypanosomiasis (Chagas Disease)

Chagas disease, caused by the protozoan *Trypanosoma cruzi*, exemplifies both the promise and challenges of repurposing triazole drugs for parasitic infections. Although standard treatments (benznidazole and nifurtimox) can cure the infection if they are administered during the acute stage, these nitroheterocyclic drugs are highly toxic and exhibit suboptimal efficacy in chronic infections, which represent the majority of diagnosed cases [58].

**Figure 4:**
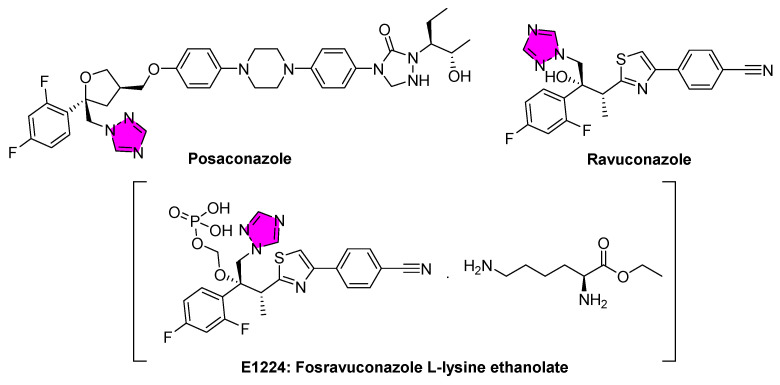
Current drug and triazole treatments evaluated in treating Chagas disease.

Since *T. cruzi* utilizes ergosterol-like sterols for membrane integrity, researchers have hypothesized that antifungal azoles might inhibit parasite sterol synthesis and clear the infection [59]. Preclinical studies have confirmed this concept; posaconazole and ravuconazole (a closely related experimental triazole) (Figure 4) have demonstrated potent anti-*T. cruzi* activity in cell culture and mouse models, even against strains resistant to nitrofuran drugs. Early investigations have shown that these triazoles could completely suppress parasitemia in mice, suggesting the possibility of a radical cure [60].

Encouraged by preclinical success, clinical trials were initiated. In the landmark Phase II STOP-CHAGAS trial [61], posaconazole (administered both as monotherapy and in combination with benznidazole) was compared with benznidazole monotherapy in patients with chronic Chagas infection. Posaconazole monotherapy produced high rates of initial parasite clearance (as measured via a negative PCR result during treatment), highlighting its trypanostatic effect. However, after six months post-therapy, parasitological relapse was common; only 13.3% of patients remained PCR-negative at 180 days, a result that is not significantly better than that of a placebo. In contrast, benznidazole monotherapy maintained efficacy, with 86.7% of patients remaining PCR-negative at 180 days. Moreover, the combination of benznidazole and posaconazole did not significantly outperform benznidazole alone in long-term cure rates (approximately 80% vs. 86%, respectively). By one year, nearly all patients who received benznidazole (with or without posaconazole) remained PCR-negative, whereas almost all patients on posaconazole monotherapy experienced relapse. These outcomes suggest that although posaconazole is highly active during dosing, it fails to eradicate chronic infections, which is likely due to persisting parasite forms in tissues that re-expand once drug pressure ceases.

A similar pattern emerged in a Bolivian clinical trial testing ravuconazole (administered as the prodrug E1224) in chronic Chagas disease [62]. Ravuconazole (E1224) induced only transient parasite clearance in chronic Chagas patients, with frequent relapses after treatment; benznidazole achieved an 82% sustained parasitological cure in 12 months, whereas high-, short-, and low-dose ravuconazole regimens yielded just 29%, 11%, and 8%, respectively (virtually identical to the 9% clearance observed with placebo). These clinical findings underscore that triazole monotherapy is insufficient for eradicating chronic Chagas disease. Contributing factors may include the parasite entering a semi-dormant state and residing in tissue niches (e.g., adipose tissue, gastrointestinal tract) where static drug levels are inadequate or even the adaptive upregulation or bypass of the sterol pathway under drug pressure. Additionally, the host’s immune response (which is critical for complete clearance) may not be sufficiently activated by triazole treatment alone.

Despite these setbacks, triazoles remain an important component of the advanced therapeutic toolkit for Chagas disease, particularly for acute infections or reactivations in immunosuppressed patients where rapid parasite reduction is needed and benznidazole toxicity is a concern. In acute Chagas models, posaconazole has been highly effective, and some evidence suggests that it clears parasites better in early-stage disease [63]. Ongoing strategies include optimizing combination therapy and adjunct drug evaluation. In the first strategy, although posaconazole combined with benznidazole did not show a marked synergistic effect in one trial, alternative dosing regimens (e.g., sequential therapy) [64] may allow a triazole to sterilize niches that benznidazoles penetrate poorly, while benznidazole eliminates persisters (i.e., a subpopulation of *T. cruzi* parasites that can survive prolonged drug treatment) via a complementary mechanism. In the second strategy, repurposed drugs such as allopurinol (a purine analog) and disulfiram (a cysteine modifier) are also under evaluation [65,66]. For instance, a clinical trial in Brazil is assessing disulfiram plus benznidazole in the treatment of chronic Chagas disease based on promising in vitro and murine data for disulfiram [67]. Although disulfiram is not a triazole, such combinations illustrate the broader effort to identify synergistic drug pairs.

Medicinal chemists are now designing triazole compounds tailored to specific parasitic targets, thereby enhancing their potency and selectivity compared to conventional broad-spectrum antifungals. The goal is to design triazoles that selectively bind *T. cruzi*’s CYP51 (or other essential parasite enzymes) [68,69,70] with higher affinity than they bind human or fungal counterparts. Research has yielded promising candidates, such as VNI, a vinyl-imidazole inhibitor that is potent against *T. cruzi* CYP51 and cures acute Chagas disease in mice [25]. Crystallographic studies have revealed subtle differences in the active site of *T. cruzi* CYP51, providing opportunities for increased selectivity and efficacy [71]. There is optimism that next-generation triazoles (or azole-related scaffolds) may eventually achieve a radical cure for chronic Chagas disease with fewer side effects than current therapies.

Benznidazole remains one of the primary drugs used to treat Chagas disease. Although it is effective during the acute phase, its efficacy wanes in chronic infections, with treatment effects lasting only up to 60 days. Additionally, benznidazole is contraindicated during pregnancy or in patients with kidney or liver failure, and side effects occur in up to 40% of treated adult patients [72]. Consequently, alternatives, such as the ergosterol biosynthesis inhibitor ravuconazole, have been explored [73,74,75]. However, due to its poor water solubility, ravuconazole was reformulated as fosravuconazole (a phosphorylated prodrug known as E1224), which rapidly transforms into ravuconazole after administration [76]. Fosravuconazole offers improved pharmacokinetics, a longer half-life, and increased water solubility and has shown excellent efficiency as an early treatment, with cure rates up to 100%. Yet, similar to posaconazole, its effectiveness in treating established infections is transient [62,77,78,79]. Combination studies, such as those conducted by the BENDITA initiative (Benznidazole New Doses Improved Treatment & Therapeutic Associations), have evaluated fosravuconazole at a dose of 300 mg once daily for the first three days, followed by 300 mg once weekly, in combination with benznidazole dosed either at 150 mg daily for four weeks or 300 mg once weekly for eight weeks [80]. Although these combinations achieved parasitological clearance after three weeks of follow-up, their efficacy was similar to that of benznidazole monotherapy. Clinical trials evaluating posaconazole monotherapy and its combination with benznidazole have also confirmed that while these triazoles are active during treatment, they fail to produce a durable long-term cure [81].

Triazole drugs for Chagas disease have shown high in vitro and in vivo activity, although their predominantly trypanostatic effects limit their ability to eradicate chronic infections. The partial successes in clinical trials, alongside extensive structure–activity relationship data, provide valuable insights that continue to guide the development of improved triazole-based therapies. Future efforts (whether through optimized combination regimens or the design of pathogen-specific triazoles) aim to achieve a radical cure with improved safety and efficacy compared to current treatments.

### 3.2. Leishmaniasis

Leishmaniasis, caused by *Leishmania* protozoa, encompasses a spectrum of diseases ranging from cutaneous leishmaniasis (CL), which causes skin ulcers, to visceral leishmaniasis (VL, kala-azar), which becomes a lethal condition if left untreated. Standard treatments, including antimonials, amphotericin B, and miltefosine, are often toxic, expensive, or compromised by logistical problems and emerging resistance. In this context, oral azole drugs have been investigated as attractive alternatives or adjunct therapies.

Since *Leishmania* synthesizes ergosterol-like sterols for membrane integrity (similar to fungi and *T. cruzi*), it was hypothesized that azoles, which inhibit lanosterol 14α-demethylase (CYP51), could disrupt parasite sterol biosynthesis and inhibit growth. Early clinical trials from the 1980s and 1990s tested ketoconazole, itraconazole, and fluconazole for CL, with mixed results [82]. Small studies have reported cure rates of roughly 50% for CL, although efficacy varied by *Leishmania* species and lesion severity, indicating molecular and biochemical differences [83,84]. For example, ketoconazole produced an 80% cure rate in VL patients treated for four weeks [85], whereas a three-month course of itraconazole for mucocutaneous leishmaniasis yielded only a 23% cure rate [86]. To address these discrepancies, an LC-MS/MS method was developed to profile sterols in various *Leishmania* promastigote species [87]. The findings, which reveal multiple intermediate sterols after azole treatment, support a branched biosynthesis pathway and suggest that CYP51 inhibition alone may be insufficient, thereby supporting the use of drug combination strategies.

Among azoles, posaconazole has shown broad activity against trypanosomatid parasites, including *T. cruzi* and several *Leishmania* species [88]. It has demonstrated efficacy in murine models against *L. donovani* (VL) and *L. amazonensis* (CL) [89]. Its potency may arise not only from target interactions but also from its structural ability to disrupt intracellular calcium homeostasis and its favorable pharmacokinetic profile [71]. For instance, both posaconazole and itraconazole have shown antileishmanial activity in the low micromolar range against *L. (Viannia) panamensis*, while comparable activity was observed against *L. donovani* (IC_50_ = 1.64 ± μM for posaconazole and 0.80 ± 0.11 μM for lansoprazole) [90,91]. In contrast, fluconazole and voriconazole displayed low activity against *L. (Viannia) panamensis*. Further case studies have supported these findings: one report detailed the successful treatment of CL caused by *L. infantum* with a regimen of 400 mg of posaconazole orally twice daily for 14 days, with no recurrence or visceral involvement for over 15 months [92]. Conversely, a combination of oral posaconazole (400 mg per day) and sensitized photodynamic therapy for CL caused by *L. aethiopica* had to be halted after 7 days due to hepatotoxicity [93].

**Figure 5:**
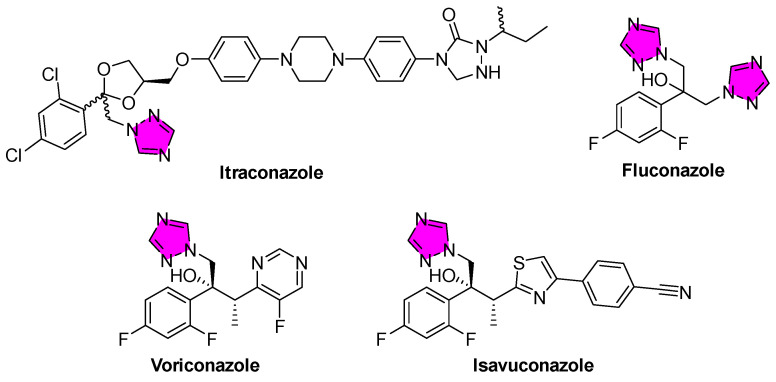
Current drug and triazole treatments evaluated in treating Leishmaniasis.

Fluconazole (Figure 5) has also shown efficacy against certain *Leishmania* species. One case report described a patient with CL due to *L. tropica* who was successfully treated with 50 mg/day for 7 days, followed by 150 mg/week for 4 weeks, achieving an 18-month relapse-free period after treatment failure with meglumine antimoniate therapy [94]. Similarly, oral fluconazole (200 mg twice daily for 8 weeks) resulted in the healing of sporotrichoid CL caused by *L. major* with excellent tolerance and no relapse after 3 months [95]. However, a randomized clinical trial comparing high-dose oral fluconazole (6.5–8.0 mg/kg/day for 28 days) with a standard antimonial protocol for CL caused by *L. braziliensis* found cure rates of only 22.2% versus 53.8%, respectively, with similar adverse effect rates (34.6% for antimonials vs. 37% for fluconazole) [96]. Moreover, a nonrandomized phase 2 trial investigating localized CL due to *L. (Viannia) guyanensis* infection demonstrated that a daily dose of 450 mg for 30 days was ineffective [97].

To combat increasing drug resistance and improve treatment efficacy, several studies have combined azoles with conventional and experimental antileishmanial agents. For example, combining posaconazole with miltefosine against intracellular amastigotes of *L. (Viannia) panamensis* showed synergistic activity, supporting a dual-target approach (*vide infra*). Similarly, a combination of amiodarone (an antiarrhythmic that blocks sterol biosynthesis, disrupts calcium homeostasis, and induces reactive oxygen species) with voriconazole against *L. major* in BALB/c mice was explored [98]. Treated mice exhibited reduced inflammatory cell infiltration, increased fibroblast activity, and enhanced collagen deposition, indicating potential therapeutic benefits. These examples underline the value of combination therapy, not only for enhancing efficacy but also for reducing individual drug doses and associated cytotoxicity.

A systematic review analyzing 37 studies (including 14 randomized trials) reported a pooled cure rate of 60–65% with azole treatment [99], which is superior to that of a placebo in some cases but still lower than the ~90% cure rate achieved with standard antimonial therapy. Notably, fluconazole showed about 88% success in treating *L. major* infections (Old World CL), while its efficacy was much lower against *L. braziliensis* or *L. tropica* (with some trials reporting cure rates below 20%). For mucocutaneous leishmaniasis (MCL) caused by *L. braziliensis*, ketoconazole and itraconazole achieved only modest results (49% cure rate). In general, adverse events with azoles were mild (7–13% of patients experienced gastrointestinal upset or elevated liver enzymes) compared to the higher toxicity of antimonials. While azoles exhibit some clinical activity in treating leishmaniasis, their efficacy as a monotherapy is inconsistent and usually inferior to standard treatments. Consequently, azoles are not recommended as sole first-line agents, except perhaps for localized *L. major* CL when antimonials are contraindicated.

Research continues to explore newer triazoles and combination strategies. The broad-spectrum triazole posaconazole has shown significant anti-*Leishmania* activity in laboratory studies [90]. Both posaconazole and itraconazole exhibited the potent inhibition of *L. amazonensis* amastigotes in macrophages with IC_50_ in the sub-micromolar values [100]. In experimental models of VL, posaconazole significantly reduced parasite loads in the liver and spleen [89]. Case reports have documented the successful treatment of Old World CL with posaconazole, especially in instances where standard therapy failed or was unsuitable [92]. Its broad-spectrum activity and long half-life make it attractive for off-label use in complex cases, such as those involving co-infections or concurrent fungal diseases [101].

Combination approaches are also under investigation. An experimental study demonstrated synergy between posaconazole and miltefosine against intracellular amastigotes of clinical *L. (Viannia) panamensis* strains, suggesting that a sterol biosynthesis inhibitor could complement miltefosine’s membrane- and signal-disrupting effects [90]. Another study showed that combining itraconazole with allopurinol (a repurposed agent for leishmaniasis) was more effective than either drug alone for the treatment of CL [102]. Such combinations not only enhance antileishmanial activity but may also allow for lower doses of individual agents, thereby reducing overall cytotoxicity. Although clinical data in humans are limited, these examples illustrate the rationale for triazole-based combination therapy: first inhibit ergosterol synthesis to weaken parasite membranes, then administer a second agent that directly kills the parasite or targets a different biochemical pathway.

At present, triazoles are not standard therapy for VL, given the availability of more effective treatments (e.g., amphotericin B, paromomycin, and combination regimens such as liposomal amphotericin B (AmBisome) with oral miltefosine). However, they remain part of the drug pipeline, particularly for cutaneous forms of VL. One particular application is in patients with concurrent fungal infections (e.g., *Histoplasma* or *Paracoccidioides*), where a single triazole could address both pathogens. Another potential role is in post-kala-azar dermal leishmaniasis (PKDL), where long-term azole therapy might offer a safer alternative to repeated antimonial treatments.

In summary, while conventional antileishmanial drugs remain the gold standard, azole antifungals have demonstrated promising antiparasitic activity with acceptable toxicity profiles. Their modest efficacy as monotherapies (coupled with species-dependent responses) has spurred ongoing research to optimize dosing schedules (e.g., higher-dose fluconazole regimens for CL) and explore next-generation triazoles such as isavuconazole [103]. Even partial efficacy with lower toxicity may be acceptable in milder cases or in combination regimens, thereby preserving more toxic drugs for refractory infections. Although they are not a panacea, triazoles contribute to a diversified therapeutic arsenal and hold promise as adjuncts or backup options as resistance to first-line agents grows.

### 3.3. Human African Trypanosomiasis (HAT)

**Figure 6:**
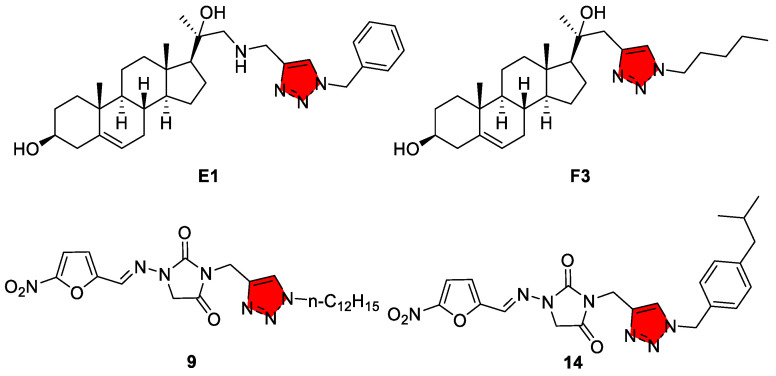
Current 1,2,3-triazole compounds evaluated in HAT.

HAT, or sleeping sickness, is caused by *Trypanosoma brucei* (sub-species *T.b. gambiense* in West/Central Africa and *T.b. rhodesiense* in East Africa). Historically, treatment involved extremely toxic organic arsenicals. In recent years, enormous progress has been made with new drugs, notably fexinidazole, a nitroimidazole approved in 2018 as the first all-oral treatment for HAT, and the combination therapy of eflornithine/nifurtimox [104]. Triazole compounds have not played a major role in HAT therapy to date. One reason is that *T. brucei* (unlike *T. cruzi*) has some differences in sterol metabolism and drug uptake that make the antifungal azoles less effective. In bloodstream-form *T. brucei*, de novo ergosterol synthesis is largely shut down, and the parasites instead scavenge host cholesterol via receptor-mediated lipoprotein uptake (an ergosterol auxotrophy that bypasses the CYP51 target of azoles) [105]. Moreover, these forms are cloaked in a dense variant surface glycoprotein coat and lack specific uptake pathways for hydrophobic azole compounds, resulting in poor drug penetration and the low intracellular accumulation of azoles [106,107]. Early studies have found that ketoconazole and fluconazole exhibit only weak activity on *T. brucei* in vitro. Additionally, HAT parasites reside in blood and the central nervous system (CNS), where drug penetration and rapid cidal activity are critical [108]; the slow-acting static effect of azoles may be insufficient. However, medicinal chemists have explored diverse triazole derivatives against *T. brucei* in vitro, and some have shown low- and sub-micromolar potency. For example, certain nitrofurantoin triazoles (Figure 6, compounds **9** and **14**) and amino steryl triazole compounds (Figure 6, E1 and F3) were reported to exhibit potent in vitro activity against *T. brucei* [70,109]. None have yet progressed to clinical trials.

In the extensive review of repurposed drugs for treating NTDs by the WHO [110], there were no azole antifungals listed for HAT, indicating that few if any clinical studies have been conducted. Instead, attention has gone to other repurposed drugs (like the cancer drug difluoromethylornithine, DFMO, which became eflornithine). Given that HAT is now close to elimination in many areas (thanks to fexinidazole and another new drug, oral SCYX-7158/acoziborole [111]), the need for triazoles in HAT is not a priority. Currently, triazoles are not part of HAT treatment guidelines, and prospects for their use remain uncertain unless a unique niche is found (e.g., a combination with fexinidazole to prevent resistance, although fexinidazole is already highly effective alone).

### 3.4. Malaria

Malaria, caused by *Plasmodium* parasites, has not traditionally been treated with triazole-containing drugs. However, significant research is underway to exploit triazole scaffolds in antimalarial drug discovery [112]. Unlike fungi or *T. cruzi*, *Plasmodium* parasites lack a complete de novo sterol biosynthesis pathway and therefore do not produce ergosterol; instead, they continuously scavenge cholesterol from host sources, such as low- and high-density lipoprotein particles and the erythrocyte membrane [113,114]. Therefore, classical antifungal azoles do not directly impact parasite survival. Consequently, new triazole derivatives are being designed to act via alternative mechanisms.

**Figure 7:**
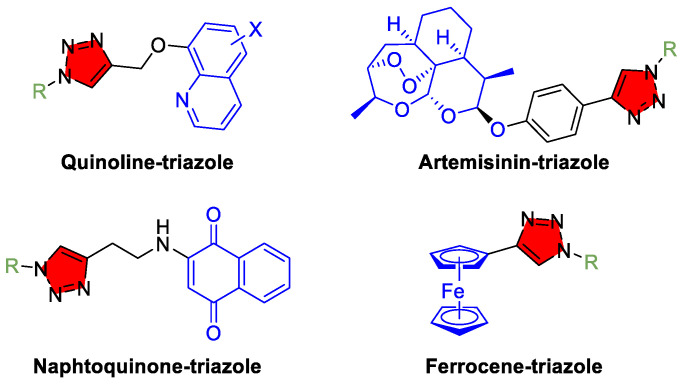
Triazole hybrid scaffolds were evaluated and exhibited promising antimalarial activity.

Medicinal chemists recognize 1,2,4-triazole and 1,2,3-triazole rings as “privileged” substructures that impart favorable properties. Recent reviews of 1,2,3-triazole-derived antimalarial scaffolds have highlighted many potent experimental compounds [47]. One strategy involves synthesizing hybrids of triazoles with established antimalarial pharmacophores (Figure 7). For example, triazole–quinoline hybrids have been developed to overcome chloroquine resistance, while others combine triazoles with artemisinin or naphthoquinones to target multiple parasite pathways [115,116,117]. Some of these hybrids exhibit low-nanomolar activity against *P. falciparum* in vitro (even against multidrug-resistant strains), and in vivo studies in mice have demonstrated parasite clearance and improved survival [48]. Another approach uses 1,2,3-triazoles as rigid linkers to construct dimeric or bis-quinoline molecules [118,119]. By tuning the length and substituents on the triazole connector, researchers have achieved enhanced potency and parasite selectivity. In one study, ferrocene–triazole–chloroquine hybrids (where the triazole tethered a redox-active ferrocene unit to a chloroquine-like moiety) showed activity below 100 nM against resistant *P. falciparum* [120,121]. Although these compounds remain preclinical, they represent next-generation antimalarial concepts. Given the emergence of resistance even to artemisinin-based combination therapies (ACTs) in some regions, new drug classes are urgently needed [122,123]. Triazole-containing molecules offer a rich area for diversification; their chemistry is amenable to modification, and the triazole ring can mimic or enhance binding interactions across a range of enzyme targets. Some analogs are being tailored to inhibit specific parasite enzymes, such as *Plasmodium* proteases or kinases [124,125].

It is important to note that no triazole drug is currently in clinical use for treating malaria, and any approved therapy may be years away. However, numerous potent leads have shown efficacy in models and even synergy with existing drugs. Among the promising candidates is DSM265, a triazole identified as an inhibitor of dihydroorotate dehydrogenase (DHODH), a flavin (FMN)-dependent mitochondrial enzyme that is critical for the de novo pyrimidine synthesis pathway in *Plasmodium* parasites. DSM265 has demonstrated efficacy against both the blood and liver stages of *P. falciparum*, along with low toxicity, a long half-life in humans, and favorable pharmacokinetics [126,127]. Several clinical trials have evaluated DSM265. In a single-dose therapy, an oral dose of 400 mg administered prior to controlled human malarial infection prevented *P. falciparum* infection in randomized trials [128,129]. Therapeutic effects were observed for up to 7 days, although parasitemia developed around 15 days post-dose in some cases. Another randomized phase 1 study identified an optimal single efficacious dose of 340 mg, with peak plasma concentrations reached between 1.5 and 4 h and an elimination half-life ranging from 86 to 118 h. The most common adverse event was headaches [130]. In a phase 2 study, in patients with *P. falciparum* or *P. vivax* malaria, 400 mg of DSM265 rapidly cleared parasitemia in all *P. falciparum* patients, while 80% of those receiving 250 mg achieved clearance by day 14. In contrast, *P. vivax* infections required higher doses (up to 800 mg) due to lower clearance kinetics. Notably, a resistance-associated mutation in the DHODH gene was observed in two of four patients with recurring infection, underscoring the potential benefit of combination therapy to minimize resistance [131]. DSM265 has also been tested in combination with artefenomel (OZ439), a promising trioxolane sharing the peroxide pharmacophore of artemisinin [132]. Clinical trials of this combination have shown high cure rates in *P. falciparum* patients, making it a promising alternative to increasingly ineffective ACTs [133,134]. The advantages of DSM265 (including its safety profile and long elimination half-life) support its development as a partner drug in a single-dose antimalarial combination treatment. Together, these efforts address critical challenges in malaria, such as drug resistance and suboptimal pharmacokinetics. Although the role of triazoles in malaria treatment is currently one of future potential, their progress exemplifies how innovative medicinal chemistry can contribute to overcoming one of the world’s deadliest parasitic diseases.

### 3.5. Toxoplasmosis and Other Protozoan Parasitic Diseases

**Figure 8:**
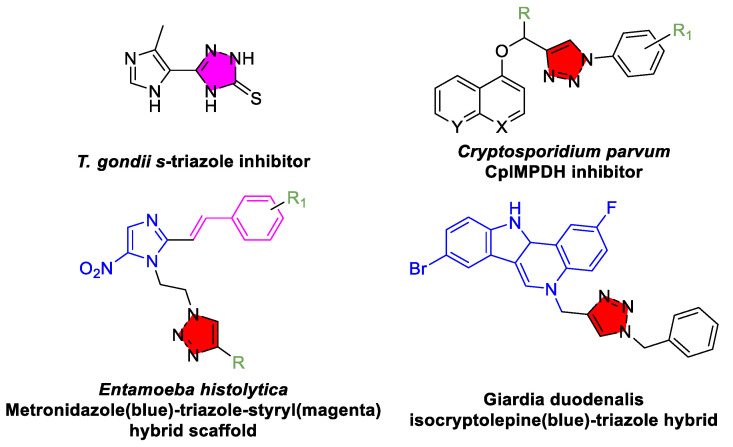
Triazoles evaluated in toxoplasmosis and other protozoan parasitic infections.

*Toxoplasma gondii*, an apicomplexan parasite like *Plasmodium*, causes toxoplasmosis, which is usually a latent infection that can reactivate in immunocompromised individuals. The standard therapy (pyrimethamine combined with sulfadiazine) often causes severe side effects, so there is interest in safer alternatives. Triazole derivatives have shown promise in this area as well. Notably, certain 1,2,4-triazole-based compounds have been found to suppress *T. gondii* growth with a markedly improved selectivity profile relative to pyrimethamine. For example, some triazoles targeting the parasite’s dihydrofolate reductase enzyme achieved high in vitro potency while sparing host cells, indicating a better therapeutic index than the current treatment (Figure 8) [135]. Moreover, because *T. gondii* synthesizes its own folates and other metabolites, triazole analogs of anti-folate drugs are being explored to overcome drug parasite resistance [136,137]. Although these approaches remain in preclinical stages, they suggest that triazoles could form the basis of next-generation anti-toxoplasmosis therapies, either as standalone agents or as adjuvants to reduce the required doses (and toxicity) of current drugs.

Triazoles have also been investigated against other protozoan infections, albeit with limited success so far. Some broad-spectrum azoles show modest activity against *Cryptosporidium* [138], *Entamoeba* [139], and *Giardia* [140], but nitroimidazoles (e.g., metronidazole) remain the drugs of choice for those parasites. Nonetheless, the chemical diversity of triazoles suggests that it may be possible to optimize compounds against these pathogens in the future. Overall, the experience across protozoan diseases shows that triazole-containing compounds are invaluable both as repurposed drugs and as novel leads, often acting via the inhibition of parasite-specific enzymes (e.g., sterol 14α-demethylase, trypanothione reductase, folate biosynthesis enzymes). The continued expansion of high-throughput screening and structure-based drug design efforts is likely to yield even more potent and selective triazole hits against protozoa.

## 4. Current Progress of Triazole-Based Therapies in Helminth Infections

Helminthic parasites (including nematodes, trematodes, and cestodes) cause diseases such as lymphatic filariasis, schistosomiasis, and soil-transmitted helminthiases, affecting over a billion people worldwide [141]. The available arsenal of anthelmintic drugs is limited, and resistance to frontline agents like benzimidazoles is an increasing concern [142]. Traditionally, triazoles have not been prominent in anthelmintic therapy because these parasites do not synthesize ergosterol, which is the target of classic azole antifungals. However, recent advances indicate that triazole derivatives can be adapted to target helminths through alternative mechanisms.

### 4.1. Schistosomiasis

Schistosomiasis remains one of the most devastating parasitic diseases worldwide, second only to malaria in terms of its global impact [143]. With over 200 million people affected and significant morbidity driven by hepatic granuloma formation and fibrosis, current treatments are largely reliant on praziquantel (PZQ) [144]. Despite its widespread use, PZQ suffers from pharmacokinetic limitations, including rapid metabolism by cytochrome P450 (CYP450) enzymes, and reduced efficacy against immature parasites [145]. Consequently, there is a pressing need for new therapeutic approaches that either enhance the activity of existing drugs or directly target schistosome biology.

A promising approach to enhancing PZQ’s antiparasitic activity is to co-administer it with triazole CYP450 inhibitors, which block the enzymes responsible for PZQ’s rapid metabolism and thereby prolong its systemic exposure and efficacy [146]. Fluconazole, a first-generation triazole antifungal, has been shown to significantly inhibit the expression of *S. mansoni* CYP450 in murine studies, particularly when administered early in the infection. In these studies, early fluconazole treatment not only reduced the transition of schistosomula to adult worms but also decreased the liver egg burden, suggesting a direct correlation between CYP450 inhibition and impaired parasite development [147]. Similarly, itraconazole (a more potent CYP3A4 inhibitor) demonstrated synergistic effects when combined with PZQ, leading to a more substantial reduction in worm load and improved liver histopathology compared to PZQ alone [148]. These findings support the concept that targeting parasite CYP450 enzymes can both enhance PZQ activity and potentially delay the emergence of drug resistance.

**Figure 9:**
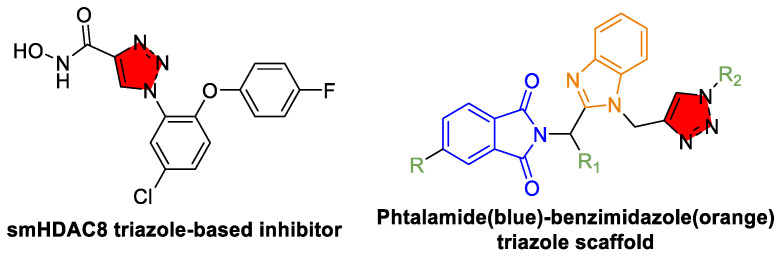
Triazole hybrids with anti-schistosomiasis activity.

Beyond their role in modulating drug metabolism, triazoles themselves have emerged as direct antiparasitic agents by targeting novel enzymatic pathways in schistosomes. One innovative approach involves the structure-based design of the triazole-based inhibitors of *S. mansoni* histone deacetylase 8 (smHDAC8). Studies have revealed that certain triazole derivatives, such as the fluorophenoxy derivative, exhibit nanomolar inhibitory activity against smHDAC8, with high selectivity over human HDACs, offering a promising lead for schistosomiasis therapy (Figure 9) [149]. By interfering with epigenetic regulation, these compounds impair parasite development and survival, thereby providing an entirely new mechanism of action that is distinct from traditional anthelmintics.

Recent synthetic advances have further expanded the chemical space of triazole-based compounds with the development of phthalimide analogs and mercaptotriazoles. Phthalimide analogs that incorporate triazole and benzimidazole moieties have been synthesized via click chemistry and subsequently evaluated against both larval and adult stages of *S. mansoni*. Many of these compounds have demonstrated potent antischistosomal activity, with favorable absorption, distribution, metabolism, and excretion (ADME) profiles and low cytotoxicity, underscoring their potential as future therapeutic agents [150]. Similarly, a recent review reported that several novel triazole derivatives have yielded compounds with significant schistosomicidal activity in preclinical models, underscoring the versatility and promise of triazole scaffolds in combating parasitic diseases [151].

Collectively, these findings highlight the multifaceted role of triazoles in advanced anti-schistosomiasis therapies. On the one hand, derivatives such as fluconazole and itraconazole enhance the pharmacokinetic profile of existing drugs like PZQ by effectively inhibiting CYP450 enzymes; on the other hand, specifically designed triazole-based inhibitors targeting novel parasite enzymes (e.g., smHDAC8) represent a paradigm shift in drug development. The emergence of diverse analogs, including phthalimide and mercaptotriazole derivatives, further expands the discovery landscape with promising in vitro and in vivo activities. Future research should focus on optimizing these compounds for improved bioavailability, selectivity, and safety, as well as exploring synergistic combinations with current therapies. Advances in structure-based drug design and chemical synthesis will continue to refine these candidates, ultimately contributing to more effective management of schistosomiasis and other parasitic diseases.

### 4.2. Other Helminthic Infections

**Figure 10:**
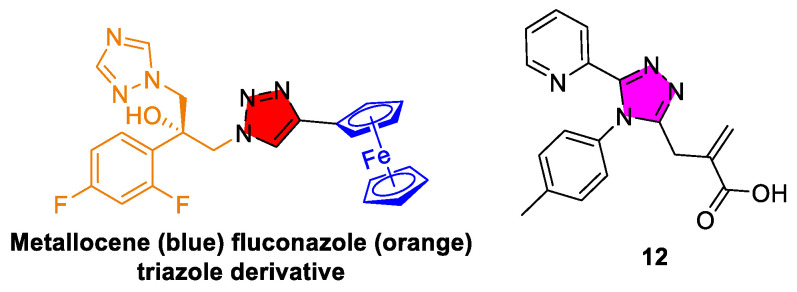
Structures of an anthelminthic organometal–triazole–fluconazole hybrid and a 1,2,4-triazole derivative with activity against various helminths.

A breakthrough approach has been the application of bioorganometallic chemistry to triazole antifungals. Recently, it has been demonstrated that attaching an organometallic fragment, such as metallocene (Figure 10), to a fluconazole scaffold creates compounds with entirely new activity profiles against helminths [152]. These organometallic triazole derivatives were highly effective in vivo against parasitic worms such as *Brugia* (the causative agent of lymphatic filariasis) and *Trichuris* (a major cause of intestinal helminthiasis), achieving cures in infected animal models. Importantly, the modified triazoles do not rely on the inhibition of sterol synthesis (inactive in helminths) but instead target pathways that are absent in humans yet essential for worm survival. This innovative strategy reveals that the triazole core can be repurposed beyond its traditional antifungal role when paired with suitable chemical modifications.

In parallel with organometallic modifications, medicinal chemists have synthesized a variety of 1,2,4-triazole-based compounds and directly assessed their activities against model nematodes [153]. A series of N^1^-substituted 1,2,4-triazole derivatives were tested for nematicidal activity using free-living nematodes as surrogates for parasitic worms (Figure 10, compound **12**). Several compounds have exhibited significant anthelmintic effects, with two analogs (LC_50_ of 2.48 μg/μL in *Rhabditis* spp.) even surpassing albendazole in potency, a standard benzimidazole anthelmintic. These compounds also showed anti-inflammatory properties in vitro, reducing pro-inflammatory cytokine production by human cells. Although this research is in its early stages, it highlights the untapped potential of triazole scaffolds in anthelmintic drug discovery. In another study, novel 1,2,3-benzotriazole derivatives were synthesized using a green, ultrasonic, and solvent-free method and subsequently evaluated for their in vitro anthelmintic activity against *Pheretima posthuma*. Notably, derivatives bearing *p*-nitrophenyl substituents demonstrated potent, dose-dependent activity that rivaled or surpassed albendazole [154].

Beyond these approaches, triazole-containing molecules might be designed to inhibit other parasite processes that are common among helminths. For instance, specific nematodes possess unique nervous system receptors or metabolic enzymes that could be effectively targeted by appropriately designed triazoles. Although no triazole-based anthelmintic has yet entered clinical trials, these proof-of-concept studies could provide a strong rationale for further investigation. While benzimidazoles remain the cornerstone of anthelmintic therapy, an intriguing research avenue could be the synthesis of triazole analogs of these compounds. For example, tribendimidine (a broad-spectrum anthelmintic used in China that features a di-benzimidazole structure) could inspire efforts to incorporate triazole rings into established anthelmintic pharmacophores, potentially enhancing efficacy and overcoming emerging drug resistance.

## 5. Resistance, Safety, and Other Considerations

Resistance, safety, and other practical considerations are pivotal as triazole-based antiparasitic therapies move toward clinical application. While drug resistance mechanisms (such as target mutations, enzyme overexpression, and efflux pump activation) are well documented in fungal pathogens, similar phenomena could emerge in parasitic organisms if these agents are deployed as monotherapies. Additionally, although triazoles generally offer a more favorable safety profile compared to traditional antiparasitic drugs, concerns such as hepatotoxicity, skin reactions, and endocrine disturbances remain. This section examines the potential for resistance, reviews key safety and toxicity issues, and discusses strategies, such as combination therapy and innovative delivery systems, that could enhance the clinical prospects of triazole compounds in the fight against parasitic diseases. All these factors have been pivotal in guiding the progress and prospects of triazole use.

### 5.1. Drug Resistance Mechanisms

Pathogen resistance to triazole drugs is a well-documented problem in fungi and could likewise emerge in parasites if these drugs are widely used. In fungi like *Candida* and *Aspergillus*, resistance to triazoles often involves mutations in the target enzyme CYP51 (altering binding affinity), overexpression of the enzyme, or the upregulation of drug efflux pumps [155,156]. Similar mechanisms could occur in protozoa. *T. cruzi* treated with sub-lethal doses of posaconazole in vitro can acquire point mutations in *Tc*CYP51, reducing drug binding (although full resistance in the clinic has not been observed, possibly because posaconazole has not been in use long) [41]. *Leishmania* spp. could potentially amplify sterol gene clusters or use efflux pumps to resist azoles [157]. A key factor in chronic Chagas treatment failure is pharmacodynamic resistance; *T. cruzi* parasites evade drug pressure not through genetic mutation but by entering dormant states or residing in tissue niches where drug levels are insufficient [158,159], resulting in persistence despite adequate dosing [160]. The use of combination therapy helps overcome this persistence by pairing drugs with different mechanisms of action, targeting both replicating and quiescent parasites simultaneously, thereby dramatically reducing the chance that any parasite subpopulation can survive treatment. This strategy has succeeded in the treatment of HIV, tuberculosis (TB), and other infectious diseases [161]. For parasites, the combination of triazoles with a fast-acting cidal drug (e.g., benznidazole or amphotericin) may kill persisters while preventing any resistant mutants from dominating. It is noteworthy that resistance to triazoles in parasites has not yet become a clinical issue, simply because these drugs are not widely used alone.

### 5.2. Safety and Toxicity

Triazole drugs, while generally safer than many older antiparasitic drugs, come with their own spectrum of side effects [162,163,164]. Common adverse effects include hepatic toxicity (elevated liver enzymes, rare hepatitis), skin reactions, gastrointestinal upset, and in some cases, endocrine disturbances (for instance, older azoles like ketoconazole can suppress steroid hormone synthesis, leading to hormonal imbalances) [165]. In the context of tropical diseases, safety is paramount because treatments often need to be administered in remote settings to patients who may have comorbidities or malnutrition [166]. Azole antifungals were originally prized for being less toxic than amphotericin B [167], making them good drug candidates for parasites. For example, they could, potentially, replace antimonial drugs in leishmaniasis to avoid antimonial cardiotoxicity. However, long-term toxicity must be monitored. In Chagas trials, posaconazole was generally well tolerated, with mostly mild adverse events, whereas benznidazole more often led to serious reactions, such as neuropathy and dermatitis [61]. These safety data suggest that incorporating triazoles into combination regimens could reduce overall toxicity.

New triazoles like isavuconazole have been optimized for safety; isavuconazole causes no QT prolongation and has fewer drug–drug interactions, which is beneficial since many patients with parasitic diseases may be on other medications (for HIV, TB, etc.). Another safety consideration is teratogenicity; azoles are generally avoided in pregnancy due to the risk of birth defects, as shown in animal studies (they affect steroidogenesis) [168]. For diseases like Chagas or leishmaniasis, treating pregnant women is a dilemma; a triazole that exhibits a better safety profile in pregnancy (if one could be found) would be an important advance against congenital Chagas disease.

As stated before, pharmacovigilance data on triazole antifungals have shown patterns of hepatotoxicity and skin reactions that guide monitoring. For instance, United States Food and Drug Administration (FDA) Adverse Event Reporting System (FAERS) analyses indicate that liver injury is a class effect, and rarer events like Stevens–Johnson syndrome or heart failure have been reported with certain azoles [36]. As triazoles are repurposed, clinicians must remain vigilant for these effects in new patient populations. Dose adjustments may be needed in those with coexisting liver disease. Encouragingly, clinical trials of triazoles for protozoal infections have not revealed any novel toxicities, and their safety profile remains consistent with that observed in antifungal applications.

In all these cases, triazole scaffolds provide a versatile platform that researchers can modify to target specific parasites or improve on existing drugs. The diverse “anti-infective spectrum” of triazoles (antiviral, antifungal, antibacterial, antiparasitic) is a double-edged sword; it means that any new triazole drug must be assessed for off-target effects and resistance in multiple contexts (for instance, if a triazole used for treating parasites also affects human flora, those risks need to be managed).

### 5.3. Regulatory and Implementation Prospects

From a prospect’s standpoint, some triazole applications are moving forward in the development pipeline. For example, fosravuconazole for mycetoma has reached phase II/III trials [169,170]. If approved, it could validate the strategy of deploying advanced triazoles in neglected diseases. For Chagas disease, despite the disappointing monotherapy results, posaconazole or ravuconazole might still have a role if employed in shorter-course adjunct therapy [171], perhaps to improve acute phase outcomes or in post-treatment management to ensure sterility (akin to how rifampicin is added in the short term in some TB regimens to prevent relapse) [172]. Any such use would require careful clinical trials. Drug cost is another factor; posaconazole is expensive, but as patents expire or generic production begins, cost barriers could fall, making these drugs more accessible in endemic countries [14].

### 5.4. Innovative Delivery Systems

Prospects for triazoles also include innovative formulations. Long-acting injectable formulations or the nanoparticle encapsulation of triazoles could provide sustained therapeutic levels, which is crucial for diseases like Chagas where long treatment courses are needed [173]. Research into the intranasal delivery of triazoles for cerebral parasitic infections, as well as topical triazole creams for cutaneous leishmaniasis, is ongoing and could expand triazoles’ use if successful [174].

### 5.5. Environmental and One Health Considerations

Interestingly, the widespread use of azole fungicides in agriculture has led to azole-resistant fungi that affect humans [175]. From a One Health perspective (i.e., a holistic approach that sustainably balances and optimizes the health of humans, animals, and ecosystems), a parallel concern arises if triazole antiparasitics are used in livestock (e.g., to treat parasitic infections in cattle), since they could select for resistance in parasites that also infect humans or persist in environmental organisms. It is a minor consideration now, but it underscores the fact that that stewardship will be important if triazoles become common antiparasitic drugs [176].

## 6. Recent Trends and Future Prospects

The integration of triazole chemistry into antiparasitic drug development has accelerated in recent years. A key trend is drug repurposing, wherein existing triazole-containing drugs (mostly developed as antifungals) are tested against protozoan and helminth parasites. This approach has identified multiple drug candidates. For example, the azole class yielded many of the top anti-*T. cruzi* leads in phenotypic screens [23]. Repurposing is attractive for its lower cost and shorter development timeline [14,23,177], but as seen with posaconazole, pharmacodynamic differences between fungal and parasitic infections can limit success. Consequently, repurposing efforts are now often coupled with combination therapy or optimized dosing strategies to maximize efficacy. The failure of posaconazole alone in chronic Chagas disease, for instance, has shifted focus to using azoles alongside standard nitro drugs in hopes of achieving synergistic parasite clearance [61].

Another prominent trend is the design of molecular hybrids and conjugates. By covalently linking a triazole moiety to another bioactive scaffold, researchers create single agents with dual functionality or enhanced pharmacokinetics. The success of artemisinin–triazole hybrids in malaria models is a prime example of this strategy’s potential [115]. Similar hybrid approaches are being explored for kinetoplastid diseases (e.g., triazole–quinoline hybrids effective against *T. cruzi* and *Leishmania*) and even for helminths [28]. For instance, triazole-based peptide conjugates are under investigation to improve drug delivery specificity [178]. This hybridization concept aligns with a broader medicinal chemistry trend of developing multi-target or multi-stage drugs to preempt drug resistance.

On the structural biology and computational design fronts, advances in parasite genomics and protein structure determination are guiding the rational design of triazole inhibitors. As more parasite enzymes and receptors are structurally characterized, the virtual screening of triazole libraries and AI-driven lead optimization are expected to play a growing role in discovering the next generation of antiparasitic triazoles [179].

Looking ahead, a major goal is to achieve clinical translation of the most promising triazole leads. This will require optimizing DMPK profiles to ensure adequate drug exposure at sites of infection (e.g., in cardiac tissue for Chagas or within macrophages for leishmaniasis) and minimizing off-target effects. Many triazoles can interact with human cytochrome P450 enzymes; therefore, mitigating potential toxicity and drug–drug interactions will be critical. Encouragingly, some next-generation candidates are moving forward; for instance, fosravuconazole has reportedly entered phase 2 clinical development for eumycetoma in Sudan, aiming to overcome itraconazole in combination with surgery shortcomings, as suggested in recent studies by the Drugs for Neglected Diseases initiative (DNDi) [169].

Triazoles have evolved from their original role as antifungal agents to become versatile components of antiparasitic therapy. Strategic repurposing (combined with innovative medicinal chemistry approaches, such as click-derived analogs, hybrid molecules, and organometallic modifications) and target-focused design have significantly expanded the triazole repertoire for combating parasitic diseases. The advances in treating malaria, leishmaniasis, Chagas disease, and helminthiasis illustrate both the potential and the challenges associated with triazole-based drugs. Interdisciplinary efforts that bridge synthetic chemistry, parasite biology, and clinical medicine are paving the way for triazoles to play an increasingly important role in advanced therapies for parasitic infections. This progress exemplifies how refining and reimagining existing chemical scaffolds can yield fresh solutions to age-old global health problems.

The future of triazoles in parasitic disease therapy appears cautiously optimistic as these agents transition from a cornerstone of antifungal treatment to a promising frontier in antiparasitic drug development. They offer clear benefits, including the convenience of oral administration, broad mechanistic potential, and generally good tolerability, yet they also face challenges in achieving complete cures and preventing resistance. Rather than serving as standalone cures, current trends favor their use as critical components of combination therapies or as scaffolds for developing new multi-target agents. The versatility of the triazole ring, whether in its 1,2,4- or 1,2,3-isomer form, confers metabolic stability and the ability to engage in diverse binding interactions, which have been harnessed to inhibit crucial parasite enzymes and to construct hybrid molecules that target multiple pathways.

We performed an extensive review of key databases (DrugBank [180], Google Scholar [181], PubChem [182], and ChEMBL [183]), which revealed that the 1,2,3-triazole scaffold has been widely explored in the context of early-stage antiparasitic research. Despite this extensive evidence of activity and synthetic diversity, to the best of our knowledge, there are currently no 1,2,3-triazole-based drugs in advanced clinical stages against parasitic diseases. This gap may be partly due to the explosion of synthesis initiated after Sharpless’s breakthrough in 2001, a development that has transformed the field in less than 25 years. Nevertheless, the widespread use and documented bioactivities of the 1,2,3-triazole scaffold, as evidenced by our findings and the literature, suggest that it will eventually progress to advanced clinical development as a promising antiparasitic therapy.

In diseases like Chagas, repurposed 1,2,4-triazole antifungals, such as posaconazole and ravuconazole, have demonstrated strong antiparasitic activity, although, as monotherapies, they have not achieved sustained cures in chronic infections; this has advanced our understanding and spurred efforts to integrate triazoles into combination regimens or use them in acute disease settings for improved outcomes. Similarly, in leishmaniasis, azoles have shown moderate efficacy. Triazoles remain investigational for conditions such as HAT and represent a rich pipeline of next-generation antimalarial candidates. Preclinical studies have demonstrated that combining triazoles with other drugs can produce synergistic effects, a concept that is supported by experiences such as the STOP-CHAGAS trial, in which combination strategies achieved promising initial parasite clearance despite challenges in sustaining long-term synergy. Moreover, the need for continual innovation in triazole chemistry is driven by concerns over pathogen resistance, which can arise through target modification or efflux, as well as by safety issues including hepatotoxicity and endocrine disruption; advances leading to safer agents like isavuconazole suggest that next-generation triazoles with improved binding and pharmacokinetics are on the horizon. With some triazole-based innovations, such as the aforementioned fosravuconazole for mycetoma, already in late-stage development and the potential for regulatory approvals for treating NTDs, the integration of triazoles into modern medicinal chemistry epitomizes a synergy between old and new in medicinal chemistry: an old heterocycle scaffold being repurposed and reinvented through modern techniques (such as click chemistry and structure-based design) to tackle ancient scourges of humankind, parasitic diseases. Progress so far has experienced both successes and setbacks, but it has undeniably expanded the therapeutic landscape. The prospects ahead involve triazole-containing drugs, whether as repurposed agents, novel analogs, or integral parts of combination therapies, playing an increasingly significant role in advanced therapies for parasitic diseases. Through interdisciplinary efforts bridging chemistry, parasitology, and clinical medicine, the full potential of triazoles can be realized, contributing to the global fight against these diseases that have long plagued the tropics (and beyond). Each new triazole optimized for a parasitic target brings us a step closer to safer, more effective, and more accessible treatments, ultimately moving the needle toward disease elimination and better health for affected populations.

## Data Availability

Not applicable.

## Abbreviations

The following abbreviations are used in this manuscript:

ACTs	Artemisinin-based combination therapies
ADME	Absorption, distribution, metabolism, and excretion
CL	Cutaneous Leishmaniasis
CYP3A4	Cytochrome P450 3A4
CYP51	Cytochrome P450 51
CuAAC	Copper(I)-catalyzed azide–alkyne cycloaddition
DFMO	Difluoromethylornithine
DHODH	Dihydroorotate dehydrogenase
DNDi	Drugs for Neglected Diseases Initiative
DSM265	Experimental triazole inhibitor of dihydroorotate dehydrogenase
E1224	Fosravuconazole prodrug (phosphorylated form of ravuconazole)
FAERS	FDA Adverse Event Reporting System
FDA	Food and Drug Administration
HAT	Human African trypanosomiasis
HIV	Human immunodeficiency virus
IC_50_	Half maximal inhibitory concentration
LC-MS/MS	Liquid chromatography–tandem mass spectrometry
LC_50_	Lethal concentration 50
MCL	Mucocutaneous Leishmaniasis
NTDs	Neglected tropical diseases
PDB	Protein Data Bank
PCR	Polymerase chain reaction
PLIP	Protein–ligand interaction profiler
PZQ	Praziquantel
SCYX-7158	Acoziborole (experimental drug for HAT)
smHDAC8	Schistosoma mansoni histone deacetylase 8
VL	Visceral Leishmaniasis
VNI	Vinyl-imidazole inhibitor
